# Introduction to Computational Proteomics

**DOI:** 10.1371/journal.pcbi.0030114

**Published:** 2007-07-27

**Authors:** Jacques Colinge, Keiryn L Bennett

**Affiliations:** Whitehead Institute, United States of America

## Introduction

Proteomics is defined as the protein complement of the genome and involves the complete analysis of all the proteins in a given sample [1,2]. Several technologies are involved, and numerous questions concerning the proteins are addressed. What proteins are contained in a biological sample? At what concentration do the proteins exist? How do protein expression levels alter in different samples? What are the posttranslational modifications (PTMs)? Where in the cell [3] or an organism [4] are the proteins localised? How do the proteins interact with other proteins or molecules [5,6]?

The following discussion concentrates on computational aspects of protein identification. Characterization (identification of protein modifications), quantitation, and sample comparisons are also discussed briefly.

**Figure 1:**
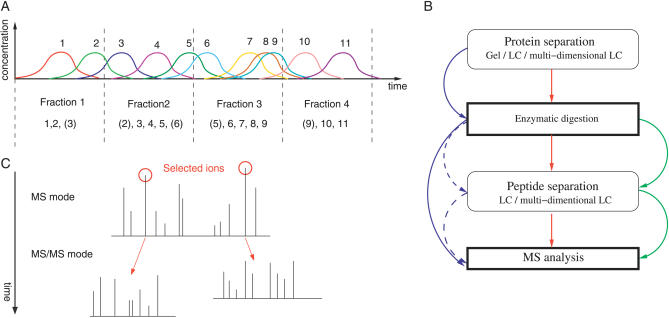
Steps in Sample Analysis by Proteomics (A) Sample complexity reduction via an LC column. This is applicable to both proteins and peptides. It is possible to collect fractions at fixed or variable time intervals to obtain a series of less complex samples; however, direct MS analysis is also an option. The figure illustrates how peptides/proteins 1–11 are fractionated. (B) Major steps in “bottom-up” proteomics and combinations thereof. Optional steps and essential steps are in rounded and bold rectangles, respectively. Green represents *shotgun peptide sequencing* entire sample digestion followed by multidimensional LC separation of peptides. Blue represents the classical gel approach, with or without (dashed arrows) peptide LC. Red combines protein and peptide LC. (C) Data-dependent MS/MS analysis. Here, ESI of a liquid sample and alternation of the instrument between MS and MS/MS modes is illustrated. The data generated is a sequence of peptide experimental *m/z* associated with the corresponding fragments *m/z*. The complete analysis is named an LC-MS run.

A typical proteomic experiment involves the analysis of complex samples, i.e., containing many proteins at varying concentrations [7]. Most of the currently available technology for identifying proteins from biological samples simply cannot contend with the complexity, and the majority of the low-abundance proteins are not observed. There are, however, a number of methods to separate the proteins contained in the original sample to obtain a simpler sample set that is amenable to in-depth analyses. Typical technologies are electrophoretic gels [8] and liquid chromatography [9] (LC) (see Figure 1A).

A dominant and well-practiced technique in proteomics is referred to as the “bottom-up” approach. Proteins are digested into peptides (smaller components of the protein) by a proteolytic enzyme, e.g., trypsin. Analysis of the peptides is achieved by mass spectrometry (MS), and, from the data generated, the peptides (and subsequently the proteins) can be identified. The resultant mixture of peptides obtained from the digestion of several proteins is often highly complex, and a degree of separation can be achieved by peptide LC. Possible combinations of separation techniques are illustrated in Figure 1B.

Mass spectrometers comprise three main components: an ion-source, a fragmentation cell, and a mass analyzer. Each component is essentially independent from the others, and as such it is possible to combine the different technological aspects to produce different types of mass spectrometers. To measure its molecular mass, a molecule must be ionised. This occurs in the ion source of the mass spectrometer. The source can be based either on electrospray ionization [10] (ESI), which is therefore appropriate for liquid samples; or on matrix assisted laser desorption ionization [11] (MALDI), which is appropriate for samples that have been mixed with a matrix and crystallized on a metallic plate. The most common types of mass analyzers used in proteomic laboratories are (i) ion trap (IT), where the radio frequency of the trap is varied and the ejected ions are detected; and (ii) time-of-flight (TOF) analyzers, where the time required for an ion to “fly” through an electric field–free region of the instrument is recorded and correlated to the mass of the ion. Most current instruments include a fragmentation cell that uses an inert gas to break the peptides by collision-induced dissociation (CID). A fragmentation cell, however, is not always present (see next section), or fragmentation can occur “spontaneously” (in-source and post-source decay). All mass spectrometers do not measure mass directly, but rather the mass-to-charge ratio. Hence the measurements obtained are dependent on the charge state(s) of the molecule.

## Peptide Mass Fingerprinting

**Figure 2:**
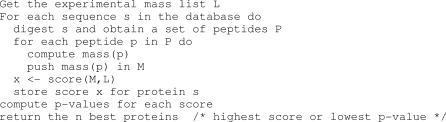
Peptide Mass Fingerprinting Database Search Algorithm

Separation of proteins by 2-D gel electrophoresis produces numerous spots that essentially contain one dominant protein. It is possible to enzymatically digest the protein in situ and measure peptide masses by MS. Historically, mass measurement of the digested proteins was initially performed with a matrix assisted laser desorption ionisation time-of-flight (MALDI-TOF) instrument. The ions generated by MALDI-TOF-MS are predominantly singly charged; therefore, the mass of the peptide can be easily calculated. Once the mass spectrum that is obtained has been signal-processed, a list of peptide experimental masses is generated (see the next section, Peak Detection). This mass list is also referred to as the experimental spectrum. The data generated can be searched against a protein database by comparing each protein sequence with the experimental peptide mass list. The comparison requires computation of a theoretical mass spectrum by digesting the sequence in silico and calculating theoretical peptide masses. A score is computed to measure the correlation between experimental and theoretical data. The highest-scoring sequence is assumed to be correct [12–14] (see Figure 2). In addition to the score, it is sometimes possible to estimate a *p*-value for the match between experimental and theoretical data.

The procedure described in the previous paragraph, named peptide mass fingerprinting (PMF), relies on a site-specific enzyme that cleaves at precise locations in the proteins. For example, trypsin cleaves after both lysine and arginine residues, provided the next amino acid in the sequence is not a proline residue.

Conceptually, PMF is straightforward and clearly introduces the principle of MS data identification by database searching. Nevertheless, when searching large databases, or when the number of available peptides is limited, the risk of false positive identification becomes increasingly higher. The presence of modified (PTMs) or incompletely cleaved peptides further reduces PMF data specificity. Moreover, the experimental design may not be amenable to 2-D gel analysis, and as such the assumption that one protein is analyzed at a time is no longer valid. Therefore, an MS technology that allows more than single protein analysis and provides additional information on each peptide would be a marked improvement over PMF.

Two programs and a parameter file (Text S1–S3) and a mass list (Text S4) are provided to illustrate the implementation of a simple PMF search algorithm.

## Peak Detection

The program extracting a list of masses from an experimental spectrum (usually provided by the MS instrument manufacturer) is essential in the identification of MS data. The performance of the algorithm and the quality of the data produced play an important role in both database searching and de novo sequencing. There are several methods to extract masses that range from straightforward local maximum detection to sophisticated wavelet analysis.

**Figure 3:**
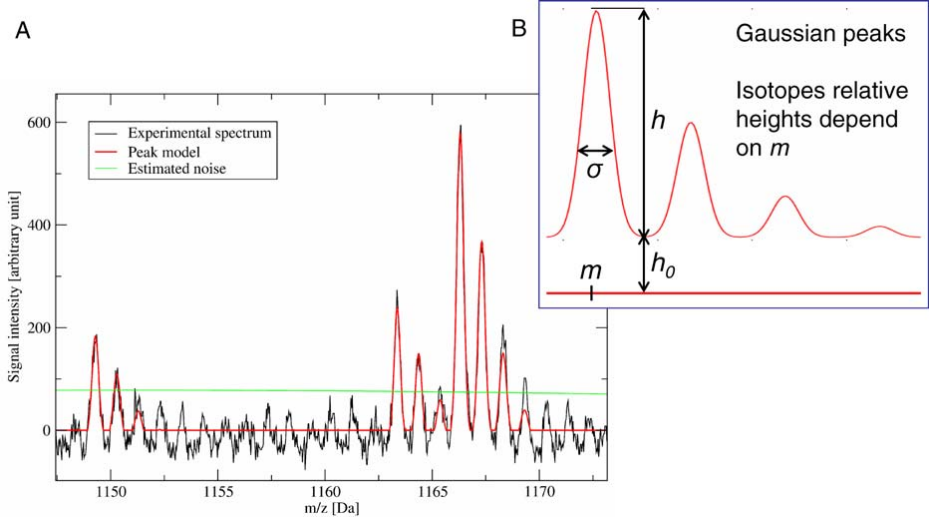
Peak Detection (A) Shown in this magnified region of a MALDI–PMF spectrum are the signals generated by peptides. The spectrum is acquired from a mixture of several peptides. Multiple copies of each peptide are present simultaneously. Multiple copies of a peptide (each detected with a small mass error) result in the essentially Gaussian shape of the peaks. Each copy comprises atoms containing different isotopes. Finally, one peptide yields several peaks with relative intensities that match the relative probabilities of the observed isotopes. The monoisotopic peak, i.e., the first peak, is relevant for mass computation. It is noteworthy to mention that the signal is noisy and the sampling limited. Shown in red is a model of a complete peptide signal fit to the experimental data. From the model location *m,* the mass can be directly deduced and detection of isotopes as additional peptide masses is avoided. The green line is an estimation of the local noise level. (B) Principle of the model.

In Figure 3, a successful method for MALDI-PMF peak detection is illustrated. Successive isotopic peaks are identified simultaneously by fitting a global model. Limited resolution of certain instruments and multiple charge states observed in ESI-MS cause additional difficulties. Such issues can make peak detection more problematic than that suggested in Figure 3.

## Tandem Mass Spectrometry

From the point of view of data processing, tandem mass spectrometry (MS/MS) can be introduced as an additional level to mass fingerprinting. There are ways that peptides can be broken into smaller molecules (fragments). As the fragmentation process is governed by certain rules, the set of fragment masses constitutes specific data. By taking advantage of such peptide-specific mass sets, it is possible to identify the peptides.

**Figure 4:**
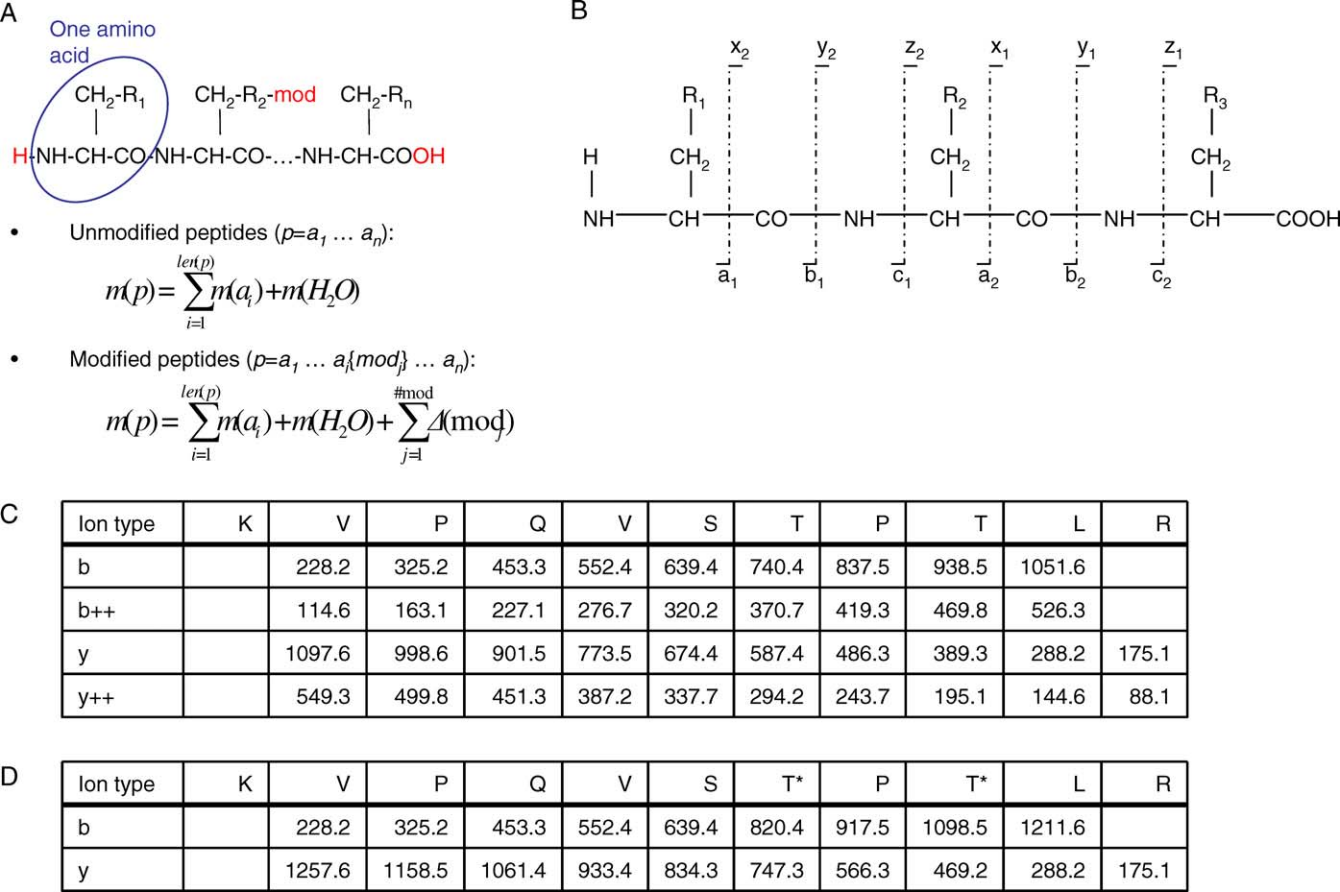
Peptide Theoretical Mass Computation and Fragmentation (A) As illustrated, the peptide atomic composition is dependent on the residue R_i_ and on fixed atoms (H_2_O). Therefore, once the peptide sequence is known, it is possible to sum the mass of each amino acid and add the mass of a water molecule to determine the theoretical mass of the peptide. If some amino acid residues are modified, mass shifts are added to the unmodified peptide mass. (B) Peptides fragment at specific locations named a,b,c,x,y,z. N-terminal fragments are termed a*_i_*,b*_i_*,c*_i_*, where *i* denotes the number of amino acids in the fragment. Similarly, the complementary C-terminal fragments are termed x*_n_*_−1_,y*_n_*_−1_,z*_n_*_−1_, *n* is the peptide length. (C) Example of fragment mass computation. (D) The same example as in (C) with phosphorylated threonine residues (+79.9663 Da). Note that all fragment ions including the ion with one or two threonine residues are shifted in mass once or twice, respectively.

**Figure 5:**
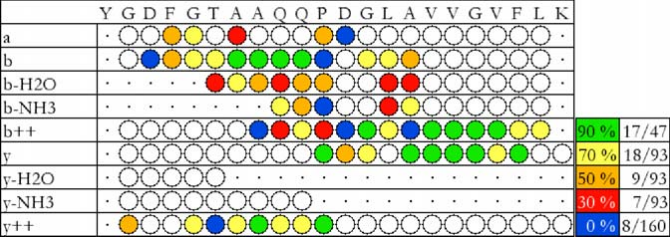
Peptide Match Match of an experimental spectrum with a peptide sequence. All theoretical fragment masses (within a given mass tolerance) observed in the experimental data are represented by a coloured disk. As is often the case, it is clear that not all fragment types are detected. Some neutral losses are not possible depending on the fragment amino acids (shown by a dot). Structural properties of the match are apparent, i.e., consecutive fragment ion matches (albeit with “holes”); and more intense b and y fragments (indicated by the colour, peak intensity relative order scale on the right with relative count of matched peaks).

**Figure 6:**
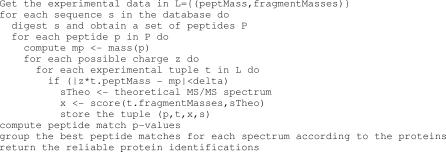
MS/MS Database Search Algorithm In this simplified MS/MS search algorithm, we assume that the peptide charge states are unknown and that all possible values (1–4 typically) need to be assessed. In practice, charge state determination is dependent on instrument mass resolution. Additionally, it is common that the charge is known for some, but not all, peptides.

The peptide fragmentation process can be induced in many ways [15,16], e.g., by collision with an inert gas. A detailed explanation of the peptide fragmentation process is not within the scope of this paper. Nevertheless, briefly, two molecules (prefix and suffix) are created when a peptide is fragmented. As the fragmentation process can occur on multiple copies of the peptide, many (albeit not all) prefix and suffix ions are observed. Fragmentation, however, is not possible throughout the entirety of the peptide. Only well-defined ion types (a,b,c,x,y,z) are generally observed (see Figure 4A and 4B). Depending on the amino acid composition, some fragments can lose a water or ammonia molecule (a neutral loss) that results in b-H_2_O, b-NH_3_, y-H_2_O, etc., fragments. Consequently, given a peptide sequence, there are rules for computing theoretical fragment masses, and it is possible to compare theoretical and experimental MS/MS spectra during a database search (see Figure 5). Based on the peptides identified, protein identification can be deduced by mapping the observed peptides onto the protein sequences (see Figure 6). A program (Text S5) and a mass list (Text S6) are provided to illustrate the implementation of MS/MS database searching.

The ability to identify individual peptides enables the analysis of complex peptide mixtures, as the peptides can be readily separated by LC. As was the requirement for PMF, with this approach it is no longer necessary that all peptides from a protein be contained within a single spectrum. A standard procedure is to analyze a liquid sample with an LC-ESI-MS/MS instrument in data-dependent mode. That is, the peptides are separated by an LC column, and the liquid phase containing the peptides is continuously introduced and ionized in the source of the mass spectrometer. The instrument in effect then “scans” the fluid for peptides by alternating between MS and MS/MS acquisitions. Peptide masses are acquired in MS mode, and a predefined number of the most intense peaks are selected for fragmentation in MS/MS mode. The instrument then returns to MS mode, and the alternating cycle continues. See Figure 1C.

The flexibility obtained by the analytical procedure described above is exploited in shotgun proteomics [17]. Here, protein separation is not performed and the sample is digested in its entirety. The complete digest is then analysed by multidimensional peptide LC. Peptides from one single protein are dispersed over many LC fractions.

## MS/MS Scoring Functions

The comparison of theoretical and experimental MS/MS spectra is performed by a scoring function, and the score (ideally complemented by a *p*-value) is used to recognize the correct peptide from a database. Reliable peptide identifications can then be considered for protein identification.

The most intuitive notion of score is provided by shared peak count (SPC), i.e., the number of masses shared by experimental and theoretical spectra within a given mass tolerance *δ*. In practice, SPC does not perform well. All matched masses are weighted identically, although some are more reliable (i.e., informative) than other masses. For example, peptide fragmentation creates several fragment ion types (see Figure 4B), and some are detected more frequently than others. Therefore, the presence/absence of frequently observed fragments should contribute more to the score compared with fragments that are seldom observed. SPC also suffers from other limitations. Some “global” properties of correct matches are ignored, e.g., the series of consecutive fragments detected and the peak intensities (see Figure 5). A high-quality scoring function should capture some of the properties that characterize a correct identification; namely, to match as many reliable fragments as possible (typically b/y), to explain the most intense peaks, and to contain some global pattern. Presented below are the scoring functions of three well-accepted search engines.

SEQUEST [18] (Thermo Scientific, http://www.thermo.com) scoring function is heuristic in nature. In fact, SEQUEST uses two scoring functions. The initial one is used to rapidly determine the best 200 peptide candidates for each MS spectrum, and a second function rescores the 200 hits. The computation of the initial score *Sp* is performed by the formula (simplified, no immonium ions)

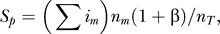
where ∑*i_m_* is the sum of matched fragment ion intensities, *n_m_* is the number of matched fragment ions, *n_T_* is the total number of fragment ions. The factor *β* rewards the continuity of a fragment series (contiguous matches); *β* has an initial value of 0 and is incremented by a small value each time successive b or y ion matches are found. See the SEQUEST patent [19] for possible *β* values. Obviously, *S_p_* score comprises the properties of correct matches as described above. Note also the division by *n_T_*, the role of which is to avoid artifactual high scores with long peptide sequences and/or long experimental mass lists.

The final SEQUEST score computation is achieved by converting the theoretical fragment masses into an artificial spectrum and by computing a cross-correlation (*a* * *e*)(*t*) between the artificial spectrum *a* and the experimental mass list *e*, with delay *t*:

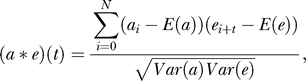
where *E*(.) denotes the mean. To take into account possible random matches and to rescale score values onto [0;1], the final SEQUEST score is defined as

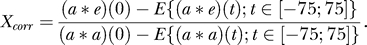



In addition to the *X_corr_* score, SEQUEST exports several other factors, e.g., *S_p_* and the difference between the best and second-best scores. Several authors have utilised this information to develop machine learning methods to detect patterns that are characteristic of correct and false matches [20–22]. These meta-scores are usually an improvement over *X_corr_*.

Mascot [23] (Matrix Science, http://www.matrixscience.com) scoring has never been published nor patented. It involves the selection of two fragment ion types, where most fragment matches are observed, and a probability-based score is computed on the basis of these two fragment types only. Experimental mass list pre-processing is also part of the Mascot algorithm. Mascot score is the negative logarithm of a *p*-value. The latter pre-processing and the selection of two fragment types are intended to obtain a more robust scoring system.

The last approach presented here is based on likelihood ratios [24]. It is assumed that the fragment matches constitute independent Bernoulli events, and the probability of these events depends on the fragment type *θ* ∈ *S* only, where *S* = {*a*,*b*,*y*,…,} is the set of possible fragment types. This probability is denoted as *p_θ_*. If *s = a_1_…a_n_* is a peptide sequence and *a_i_* the constituent amino acids, then the probability of a correct match between *s* and an experimental spectrum is estimated by taking the product of *p_θ_* for every matched fragment and of 1 − *p_θ_* for every unmatched fragment. The null-model is identical with random fragment match probabilities *r_θ_*. We find:

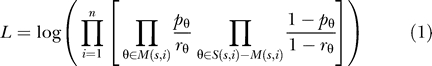



*S*(*s*, *i*) ⊂ *S* is the set of fragment types ending at amino acid *a_i_*, *M*(*s*, *i*) ⊂ *S*(*s*, *i*) is the set of fragment types matching the experimental fragment mass. *S*(*s, i*) may be a proper subset of *S* because certain fragments are not always possible depending on their amino acid composition (neutral loss). Probabilities, *p_θ_*, *θ* ∈ *S* are learnt from a set of correct matches. Probabilities of random fragment matches *r_θ_* are learnt from random peptides. Preferably, only the fragment types with probabilities *p_θ_* and *r_θ_* sufficiently different are actually used in the scoring function *L*.

**Figure 7:**
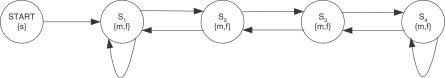
Consecutive Fragment Matches To detect sequences of consecutive fragment matches for a given type of fragment, it is possible to use a HMM. A sequence of symbols the length of the peptide is observed with alphabet letters {m,f}, m for match and f for failed match. The model topology is designed to accommodate for some missing matches: S_1_ represents a first uninformed match, whereas S_2_ and S_3_ represent matches with preceding matches.

This approach can be extended by introducing more complex models that capture additional properties of correct and random peptide matches [25–27]. A hidden Markov model (HMM) was used to model sequences of consecutive fragment matches with mismatch tolerance (Figure 7) and models similar to Equation 1 to model peak intensities and the influence of amino acid composition [27]. These scoring functions are implemented in Phenyx (Geneva Bioinformatics, http://www.genebio.com), and some performance comparisons can be found in Colinge et al. [27] and Heller et al. [28].

## Modified Peptides

It is possible that some amino acids are modified (PTMs, chemical modifications), resulting in mass shifts. Such changes in mass need to be taken into account to correctly compute theoretical MS/MS spectra. The simplest cases are fixed modifications, e.g., carboxyamidomethyl cysteine (+57.02146 Da). All cysteine residues in a protein are reduced (i.e., the disulfide bonds are broken) and the nominal amino acid mass is replaced by a shifted mass in all computations. There are also variable modifications that are not present systematically. In this case, it is necessary to compute several theoretical spectra to cover all eventualities (see Figure 4D). A common example of a variable modification is oxidation of methionine residues (+15.9945 Da). Such a modification is almost always possible and would mask peptide identifications if ignored.

In practice it is not feasible to allow many variable modifications when searching mass spectrometric–generated data against a database. Search space and time is markedly increased as is the false positive rate.

## Protein Identification

**Figure 8:**
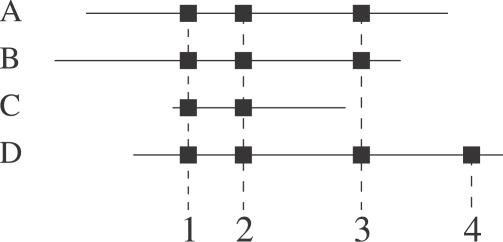
Issues in Protein Identification Complications in identifying proteins. Four proteins (A, B, C, D) are identified by four distinct peptides (black squares). Although A and B are different, it is impossible to ascertain which molecule is present, as both have been identified by the same (shared) peptides. A variation of this is shown in C. Protein D shares three peptides with A and B, and two with C, but also has a specific fourth peptide. From this information it can be concluded that D is in the sample.

Obtaining reliable peptide identifications is an essential step toward reliable protein identifications; however, some additional aspects need to be taken into consideration. Most of the problems associated with protein identification are caused by peptides shared by several proteins; see Figure 8 for an example. When two or more sequences in the database are identified on the basis of the same peptides, then it is impossible to know with certainty which molecule(s) is(are) present in the sample. This problem has been discussed extensively by A. Nesvizshkii and R. Aebersold [29].

To assign a score to a protein identification is an open question, as there are many options. A standard approach is simply to sum the highest score for each distinct peptide identified. Alternatively, it is possible to consider the multiplicity of spectra matched for each peptide to support additional evidence [30]. Not to assign a score at all is also an option, and a list of trusted proteins is the only output in that case. A classical criterion to accept a protein identification is to detect two distinct peptides above a reasonable peptide score [31]. A very small number of false positive identifications are generated by this approach.

The choice of protein database plays an important role in MS data identification. Classically, either comprehensive or curated databases have been utilised. As comprehensive databases, NCBInr (http://www.ncbi.nlm.nih.gov) and Ensembl [32] are those most frequently used, whereas commonly used curated databases are UniProtKB/Swiss-Prot [33] and International Protein Index (IPI) [34]. The latter integrates several curated databases and aims to include all alternative splice forms and active fragments. The IPI database offers a good combination of quality and exhaustiveness, which is crucial for proteomic data analysis.

## Peptide De Novo Sequencing

In the preceding sections, MS data searched against a protein sequence database was described. Situations also arise where such a database is not available or is inappropriate. A classical example is the analysis of a sample from an organism whose genome is not completely sequenced [35]. A more difficult example is the case where peptides are modified in an unexpected manner and hence are not found via the variable modifications specified during the database search. As consideration of all possible modifications is not feasible, a method that would predict part of the unmodified peptide sequence would enable the possibility of searching candidate peptides by homology before confirmation by MS/MS [36,37].

To predict the peptide sequence directly from an MS/MS spectrum is known as de novo peptide sequencing. To do this in reality is not straightforward, and prediction of short reliable sections of the sequence (so-called sequence tags) is often more realistic. The sequence tags can be used either as incomplete but reliable sequences or for searching a database by allowing mismatches. Sequence tags from several peptides from the same protein can result in specific identification of the protein.

In the early days of de novo peptide sequencing, algorithms were developed that attempted to reconstruct peptide sequences by essentially considering all amino acid combinations. Such approaches are obviously not applicable to generic problems. Currently, researchers in the field investigate graph theoretic algorithms, Markov chain Monte Carlo heuristic optimization, or HMMs. Usually, a preliminary filtering of the experimental mass list is performed to remove noisy peaks.

**Figure 9:**
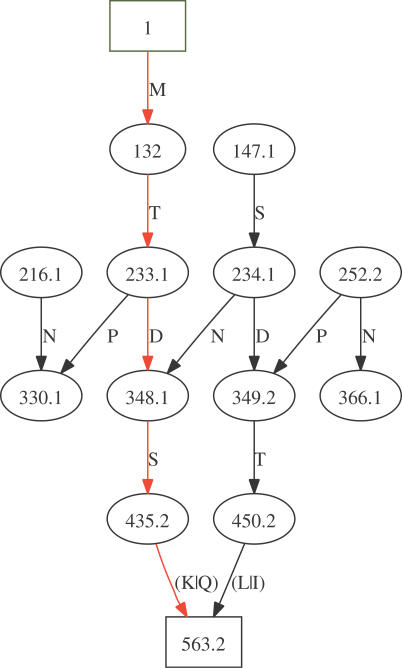
Spectrum Graph Spectrum graph of peptide MTDSK. The spectrum contains the b and y fragment ion masses plus two neutral losses and two peaks generated from noise. Only one amino acid's mass differences are accepted. Masses are complemented and interpreted as b fragments. Even in this oversimplified case, it is observed that many edges are created in addition to those that are necessary. In particular, part of the reverse sequence in the graph is observed. The graph complexity increases rapidly with real spectra and with two amino acid mass differences accepted; see also the two examples given in Figures S1 and S2.

A well-established method involves the computation of a spectrum graph *G*. Based on the masses in the experimental mass list, one vertex per mass is created; two vertices are linked provided the mass difference equals one amino acid mass within a given tolerance [38], and the edge is labelled with the corresponding amino acid (see Figures 9, S1, and S2). To contend with absent fragments, it may also be necessary to create edges for mass differences equalling two amino acids. Moreover, as it is unknown whether an experimental mass is from a C- or an N-terminal fragment, it may be necessary to complement each experimental fragment mass (peptide mass minus fragment mass) as the vertices are constructed. This general procedure can be adapted in several ways [24,39–41].

Given a spectrum, the problem of predicting the most plausible peptide sequence can be solved by finding a longest path in the spectrum graph [24,40,42]. The length of each edge is given by a scoring function that measures the fit between the additional theoretical masses yielded by the edge and the MS/MS data. Other algorithms use the spectrum graph to produce candidate peptide sequences that are progressively extended. This is typically achieved by iteratively growing and trimming a population of sequences [41]. It is also possible to combine C- and N-terminal partial sequences as obtained by a spectrum graph without computing one longest path [43].

A very different point of view is to define a scoring function and to optimize it over the space of all possible peptide sequences. The optimization is usually performed by a genetic algorithm [44,45].

A recent and innovative paper models spectral peaks as if the peaks were generated by a sequential process and hence applies a HMM [46].

Noisy peak filtering can be achieved by ad hoc methods that define noise according to a proportion of the total peaks or the total signal [39,41]. Alternatively, prediction of the type of each peak can be attempted, e.g., a,b,y fragment ion. Peaks that result in a reliable prediction can be included for further computation [47].

## Other Problems

To directly match proteomic data with genome sequences has attracted significant attention because there is the potential to complement and correct genome annotations by MS data. This potential is indeed confirmed by new findings reported by several authors [48–50].

The problem of genome searching can be approached in different ways. The most challenging case is to search MS data against a eukaryotic genome, as peptides can be coded across exon/intron boundaries. One method is to use a gene prediction algorithm to obtain protein sequences that are searched as per a standard protein database. An alternative method is to use de novo predictions and to search the predicted sequences by homology. Finally, it is possible to combine gene structure predictions and MS data searches to reveal and validate splice sites [51].

Sample comparison is essential in proteomics, and several methods have been developed to quantitatively evaluate datasets. With 2-D gels, spot volumes can provide semiquantitative information [8,52]. It is also possible to label peptides with specific reagents that alter the mass by a known value [53]. Two or more modified samples are pooled prior to LC-MS analysis. The mass shifts in the spectra indicate the origin of the peptide, and relative peak intensities provide quantitative information.

Label-free methods have been introduced that require neither 2-D gels nor peptide modification. These methods either sum all the peak intensities of a given peptide during one LC-MS experiment [54] (extracted ion chromatogram) or count the number of spectra matching the peptides of a protein [55,56]. Alternatively, it is possible to use protein chips to measure protein concentration [57].

In each case, a protein can be assigned an expression profile across samples, and techniques similar to micro-array data analysis can be applied.

Despite the great importance of PTMs for biological function, studies on a large scale are difficult [58,59]. In the context of computational analyses, comprehensive approaches toward general PTMs are difficult. Although many laboratories have undertaken detailed investigations of a specific modification in the quest to determine answers to a particular biological question, e.g., phosphorylation events in signalling pathways, most of these studies have involved manual or semi-automated annotation of the modification site(s), and data processing is more a matter of storing and visualizing. Bioinformatics has later contributed in a systems biology approach by utilising the information gained from such studies to assign function to the proteins and to reveal biological interactions.

There are also a number of interesting and important computational proteomic questions, which are considered out of the scope of this introduction, and are therefore not covered. These include protein structure elucidation via MS; glycan and lipid analysis; direct profiling of samples by MS, i.e., metabolomics. Here masses, not necessarily peptides, are detected in each sample and are comparatively analysed.

## Resources

InSilicoSpectro [60] is an open-source Perl project that implements many MS-related computations and contains numerous simple examples illustrating some of the presented concepts. Two elementary implementations of PMF and MS/MS database search in C++ are provided with example data (see Text S3 and Text S5).

Phenyx is freely available at http://www.phenyx-ms.com and Mascot at http://www.matrixscience.com. Two open-source database search engines have been developed, OMSSA [61] and X!Tandem [62]. Several public MS/MS data repositories are accessible over the Internet, including Peptide Atlas (http://www.peptideatlas.org), Open Proteomics Database (http://bioinformatics.icmb.utexas.edu/OPD), and Pride (http://www.ebi.ac.uk/pride).

## Conclusion

Proteomics plays an ever-increasing and pivotal role in biological research, and there are a range of technologies available that can generate large quantities of data. The analysis of such data opens new and challenging areas of interest for bioinformatics. In addition to the utilisation of classical methods and resources, new types of data require modelling and processing. Perhaps the best example is the mass spectrum itself, which contains continuous and discrete information simultaneously. Such issues are reflected in the difficulty of designing high-performance scoring functions and de novo sequencing algorithms.

 To provide an introduction to this fascinating field of research, we have presented general concepts of proteomics. The central problem of MS data identification by database searching has been explained at an introductory level, and should allow any interested reader to grasp the fundamental concepts of this area of research. 

## Supporting Information

Figure S1 Accepting Pairs of Amino Acid Masses in De Novo Sequencing A spectrum graph generated with the same spectrum as in the paper (peptide MTDSK) but by allowing pairs of amino acid mass differences. Observe the massive increase in complexity.
(13 KB PDF)

Figure S2 Noise in Mass Spectra Impacts De Novo Sequencing A graph obtained based on a relatively small real spectrum for the peptide LRDQLGTAK by only accepting single amino acid mass differences (all the y fragments are present). This example shows why it is important to filter mass lists for noise prior to de novo prediction, since the spectrum becomes very complex otherwise.
(45 KB PDF)

Text S1 computeMOWSEMatrix.cpp A C++ program to implement the computation of the MOWSE matrix, which is used by the MOWSE PMF scoring function.
(6 KB TXT)

Text S2 MOWSE Matrix The MOWSE matrix computed by computeMOWSEMatrix.cpp (see Text S1).
(10 KB TXT)

Text S3 pmfDBSearch.cpp A C++ program implementing a minimal PMF database search algorithm.
(18 KB TXT)

Text S4 A PMF Mass List An example mass list for PMF searching (in pkl format, SWISS-PROT ID: ENO_YEAST).
(1 KB TXT)

Text S5 msmsDBSearch.cpp A C++ program implementing a minimal MS/MS database search algorithm.
(23 KB TXT)

Text S6 An MS/MS Mass List An example mass list for MS/MS searching (in mgf format, SWISS-PROT ID: ENO_YEAST).
(22 KB TXT)

## Abbreviations

ESI: electrospray ionization
HMM: hidden Markov model
LC: liquid chromatography
MALDI: matrix assisted laser desorption ionization
MS: mass spectrometry
MS/MS: tandem mass spectrometry
PMF: peptide mass fingerprinting
PTM: posttranslational modifications
TOF: time-of-flight
SPC: shared peak count
