# Supplementary figures and images for: Introduction to Computational Proteomics
Source: PLoS Comput Biol. 2007 Jul 27;3(7):e114. doi: 10.1371/journal.pcbi.0030114 (PMC1933459; doi:10.1371/journal.pcbi.0030114)

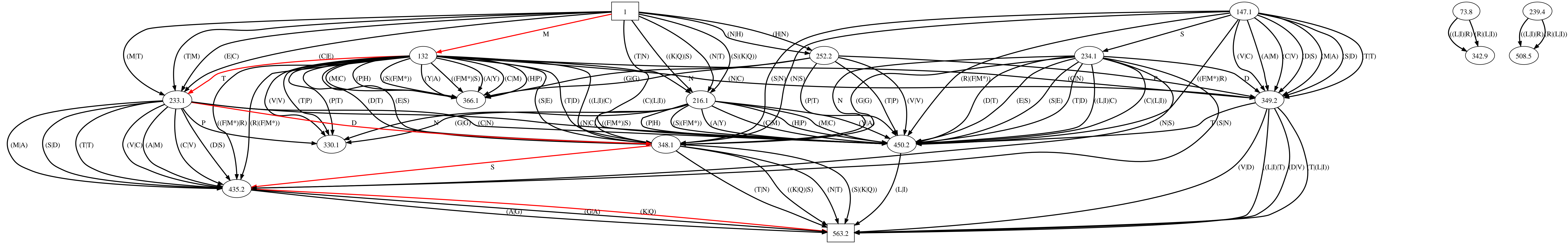

Supplement: Figure S1 — A spectrum graph generated with the same spectrum as in the paper (peptide MTDSK) but by allowing pairs of amino acid mass differences. Observe the massive increase in complexity. (13 KB PDF) [file pcbi.0030114.sg001.pdf]

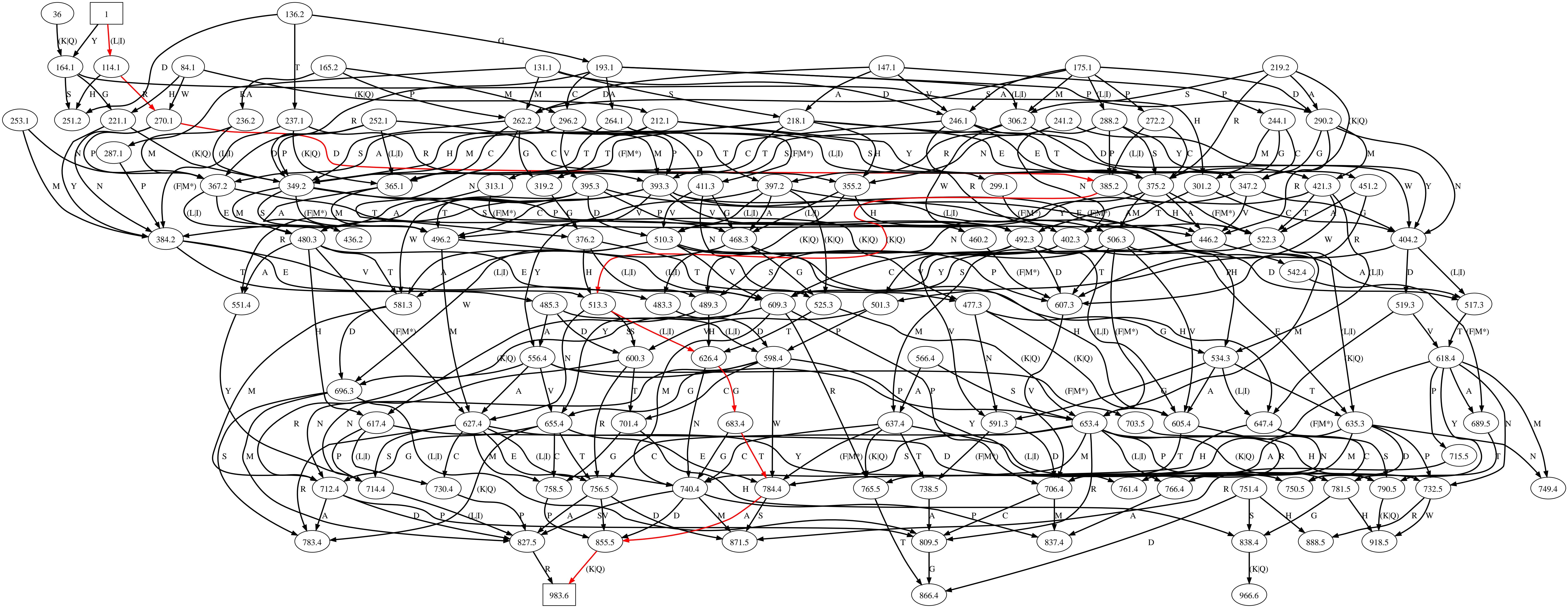

Supplement: Figure S2 — A graph obtained based on a relatively small real spectrum for the peptide LRDQLGTAK by only accepting single amino acid mass differences (all the y fragments are present). This example shows why it is important to filter mass lists for noise prior to de novo prediction, since the spectrum becomes very complex otherwise. (45 KB PDF) [file pcbi.0030114.sg002.pdf]
